# Predicting Short- and Long-Term Functional Outcomes Based on Serum S100B Protein Levels in Patients with Ischemic Stroke

**DOI:** 10.3390/jpm14010080

**Published:** 2024-01-10

**Authors:** Rakesh Jalali, Jacek Zwiernik, Ewa Rotkiewicz, Beata Zwiernik, Adam Kern, Jacek Bil, Anita Jalali, Joanna Manta, Jerzy Romaszko

**Affiliations:** 1 Department of Emergency Medicine, School of Medicine, Collegium Medicum, University of Warmia and Mazury, 10-082 Olsztyn, Poland; ewarotkiewicz@wp.pl (E.R.);; 2 Clinical Emergency Department, Regional Specialist Hospital, 10-561 Olsztyn, Poland; 3 Department of Neurology, School of Medicine, Collegium Medicum, University of Warmia and Mazury, 10-082 Olsztyn, Poland; jacek.zwiernik@uwm.edu.pl (J.Z.); beata.zwiernik@uwm.edu.pl (B.Z.); 4 Department of Cardiology and Internal Medicine, School of Medicine, Collegium Medicum, University of Warmia and Mazury, 10-082 Olsztyn, Poland; adam.kern@uwm.edu.pl; 5 Department of Invasive Cardiology, Centre of Postgraduate Medical Education, 01-813 Warsaw, Poland; jacek.bil@cmkp.edu.pl; 6 Students’ Research Group, Medical University of Warsaw, 02-091 Warsaw, Poland; s079559@student.wum.edu.pl; 7 Department of Family Medicine and Infectious Diseases, School of Medicine, Collegium Medicum, University of Warmia and Mazury, 10-082 Olsztyn, Poland; jerzy.romaszko@uwm.edu.pl

**Keywords:** ischemic stroke, functional outcome, S100B

## Abstract

Background: Ischemic stroke is one of the leading causes of mortality and disability. The neuroimaging methods are the gold standard for diagnostics. Biomarkers of cerebral ischemia are considered to be potentially helpful in the determination of the etiology and prognosis of patients with ischemic stroke. Aim: This study aimed to investigate the usefulness of serum S100B protein levels as a short- and long-term prognostic factor in patients with ischemic stroke. Study design and methods: The study group comprised 65 patients with ischemic stroke. S100B protein levels were measured by immunoenzymatic assay. Short-term functional outcome was determined by the NIHSS score on day 1 and the difference in the NIHSS scores between day 1 and day 9 (delta NIHSS). Long-term outcome was assessed by the modified Rankin Scale (MRS) at 3 months after the stroke. At the end of the study, patients were divided into groups based on the NIHSS score on day 9 (0–8 “good” and >8 “poor”), the delta NIHSS (“no improvement” ≤0 and >0 “improvement”), and the MRS (“good” 0–2 and >2 “poor”). Differences in S100B levels between groups were analyzed with the ROC curve to establish the optimal cut-off point for S100B. The odds ratio was calculated to determine the strength of association. Correlations between S100B levels at three time points and these variables were evaluated. Results: We revealed a statistically significant correlation between S100B levels at each measurement point (<24 h, 24–48 H, 48–72 h) and the NIHSS score on day 9 (R Spearman 0.534, 0.631, and 0.517, respectively) and the MRS score after 3 months (R Spearman 0.620, 0.657, and 0.617, respectively). No statistically significant correlation was found between S100B levels and the delta NIHSS. Analysis of the ROC curve confirmed a high sensitivity and specificity for S100B. The calculated AUC for the NIHSS on day 9 were 90.2%, 95.0%, and 82.2%, respectively, and for the MRS, 83.5%, 83.4%, and 84.0%, respectively. After determining the S100B cut-off, the odds ratio for beneficial effect (NIHSS ≤ 8 at day 9 or MRS 0–2 after 3 months) was determined for each sampling point. Conclusion: S100B is a useful marker for predicting short- and long-term functional outcomes in patients with ischemic stroke.

## 1. Introduction

Despite advances in diagnosis and treatment, ischemic stroke remains one of the leading causes of mortality and disability [1,2,3]. The development of diagnostic tools, especially neuroimaging methods, makes it possible to diagnose ischemic stroke with increasing certainty, determine the size of the ischemic area, and introduce more effective treatment. Unfortunately, this is also associated with an increase in costs [4,5]. Determining the size of a stroke lesion has implications for the prognosis of patients’ short- and long-term functional improvement. The commonly available computed tomography (CT) is not very sensitive in this regard and often fails to visualize early ischemic lesions. Magnetic resonance imaging (MRI) is more sensitive but has many limitations: it cannot be performed on patients with pacemakers and other metal objects, as well as in anxious patients. It is also less accessible, more time-consuming, and generates higher costs [6,7,8]. The commonly used National Institutes of Health Stroke Scale (NIHSS) was designed to determine the physical size of the ischemic area. Unfortunately, it is not very sensitive in regard to damage to the right brain hemisphere and the posterior circulation [9,10]. Therefore, in recent decades, biomarkers of cerebral ischemia have been searched for that can assist clinicians in the differential diagnosis, as well as in the determination of the etiology and prognosis of patients with ischemic stroke [11,12]. One of the biomarkers under scrutiny is S100B. It belongs to a group of small proteins with a specific calcium-binding capacity. In the central nervous system (CNS), it is secreted from astrocytes. Its biological role is to stimulate the proliferation and maturation of neurons and to promote the development of astrocytes and oligodendrocytes. In ischemic stroke, S100B is released into the blood and cerebrospinal fluid. Increased S100B levels in the extracellular space promote neuroinflammation, thus exacerbating brain tissue damage [13,14]. Since S100B has a short biological half-life, its presence in the blood indicates an active process that damages brain tissue. Significantly, S100B release appears to take place not in the infarct core (where there is a lack of perfusion that would allow its release into the blood) but in the penumbra area and the area of local brain edema as a response to the presence of adenosine and glutamate. Thus, it is hypothetically possible to control the course of ischemic stroke and formulate a prognosis for the recovery [15,16]. The results of clinical studies conducted in recent years have suggested that patients with ischemic stroke have significantly elevated blood levels of S100B. Some studies have also revealed that S100B levels correlate with the severity of ischemic stroke, as measured by the NIHSS, and may be useful in determining functional prognosis [17,18,19,20]. The aim of our study was to investigate the relationship between serum S100B levels and their changes over time and short- and long-term functional outcomes in patients with ischemic stroke.

## 2. Materials and Methods

Patients were included in the study in years 2018 to 2020 in accordance with the protocol approved by the Bioethics Committee of the Warmia and Mazury Regional Chamber of Physicians and Dentists in Olsztyn (WMIL-KB/266/2018) dated 17 May 2018, and the Clinical Research Committee of the Regional Specialized Hospital in Olsztyn, Poland. The aim of the study was to determine the role of arterial stiffness as a risk factor for ischemic stroke and the usefulness of biomarkers, including S100B protein, in assessing early and late prognosis. After obtaining consent to participate in the study, established procedures were performed for each patient. To determine S100B levels, blood was drawn three times—on admission, on the second day, and on the third day. Blood samples were collected in biochemical tubes. Then, 10 min after collection, samples were centrifuged for 20 min with a frequency of 3000 revolutions per minute. Briefly, 1 mL of the obtained serum was transferred to Eppendorf tubes and frozen to −20 °C. Samples are durable for up to 3 months in this temperature. Prior to analysis, tubes were thawed and brought to a temperature of 20–25 °C. Samples with visible turbidity were centrifuged.

All recognized procedures for treating ischemic stroke, including thrombolytic therapy, mechanical thrombectomy, or a combination of both, were performed, depending on the indication. Stroke severity was determined with the NIHSS score and was assessed on admission and on the ninth day. The change in the NIHSS scores during that time was considered to be an indicator for the short-term functional outcome. The long-term functional outcome was assessed three months following the stroke with the Modified Rankin Scale (MRS). As in other similarly designed studies, in order to facilitate a better comparison of the obtained results, patients were arbitrarily categorized into two groups based on the NIHSS score on the ninth day: NIHSS 0–8 (mild to moderate ischemic stroke) and NIHSS >8 (moderate to severe stroke). For the same reasons, patients were divided according to the MRS score after 3 months: 0–2 (no residual symptoms to slight disability) and >2 (from slight to severe disability). A routine medical history was taken, including risk factors for ischemic stroke. The type of ischemic stroke was determined with the Trial of Org 10,172 in Acute Stroke Treatment (TOAST) classification.

Serum concentrations of S100B were determined by the electrochemiluminescence immunoassay (ECLIA) method on the Cobas 6000 device, which was calibrated with S100 CalSet. The Roche Elecsys^®^ S100 kit (Roche, Mannheim, Germany) with detection limits between 0.005 and 39 μg/L was used according to the manufacturer’s instructions. PerciControl Universal PCU1 and PCU2 were used for controlling the accuracy of measurements.

Qualitative data were expressed in percentages (%) and numbers (*n*). Quantitative data were expressed as mean and standard deviation (SD). Statistical analyses were performed with Statistica software12. Mean S100B values in two groups were compared with Student’s *t*-test for paired data. Analysis of variance was used for a larger number of groups. The correlation between variables was determined with Spearman’s rank correlation coefficient, or Pearson’s linear correlation coefficient. A *p*-value ≤ 0.05 was considered as statistically significant. The bivariate tables were analyzed with Chi-square, V-square, and Chi-square with Yates’ correction. McNemar’s test was used for paired data. ROC curves were analyzed with pROC package for R. The Youden Index was used to determine the optimal cut-off point for a continuous variable. DeLong’s test for correlated curves was used to compare two AUCs. Power calculations were performed for each ROC curve separately.

## 3. Results

In total, 65 patients were included in the study. The average age was 67 years; women constituted 52% of the study group. Demographic characteristics of the group are presented in Table 1.

Changes in the NIHSS score between admission and day 9, which are the basis for further calculations, are presented in Table 2.

When correlating the S100B level < 24 h with the short-term functional outcome as measured by the NIHSS on day 9, a significant correlation between them was revealed, similarly to the remaining time points—24–48 h and 48–72 h. When correlating the S100B level < 24 h with the long-term functional outcome as measured by MRS after 3 months, a significant correlation between them was revealed, similarly to the remaining time points—24–48 h and 48–72 h. However, there was no significant correlation between the S100B level and delta NIHSS (Table 3).

**Figure 1:**
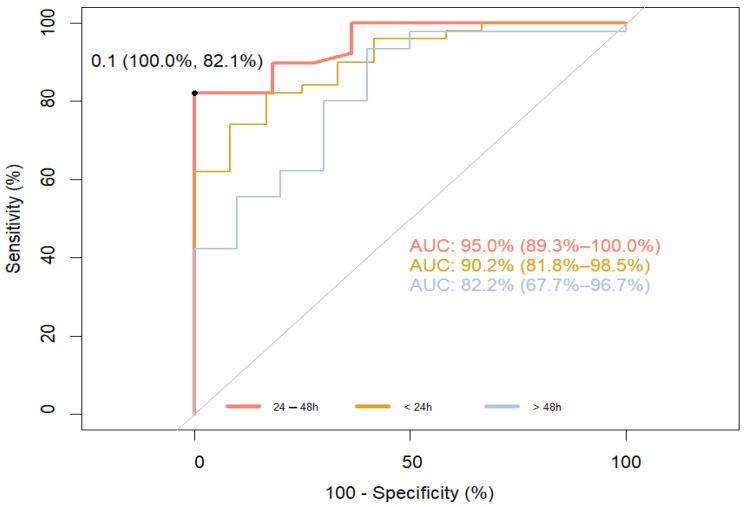
ROC curve to determine the sensitivity and specificity of S100B at different time points, predicting the outcome measured as “good” (NIHSS 0–8) and “poor” (NIHSS > 8). The bootstrap method was used to determine confidence intervals. The Youden Index was used for setting optimal thresholds.

At the end of the study, patients were divided into groups based on their NIHSS score on day 9 (0–8 “good” and >8 “poor”), the delta NIHSS (“no improvement” ≤0 and >0 “improvement”), and the MRS (“good” 0–2 and >2 “poor”). The ROC curve analysis revealed that the cut-off value of S100B in each sampling point was 0.100 μg/L. For the ROC curve to determine the sensitivity and specificity of S100B at different time points, predicting the outcome measured as “good” (NIHSS 0–8) and “poor” (NIHSS > 8), the AUC was 95.0% (89.3–100.0%) for time point 24–48 h (sensitivity 82.1, specificity 100.0%). For other sampling points, the AUC was 90.2% (<24 h) and 82.2% (>48 h), respectively (Figure 1).

**Figure 2:**
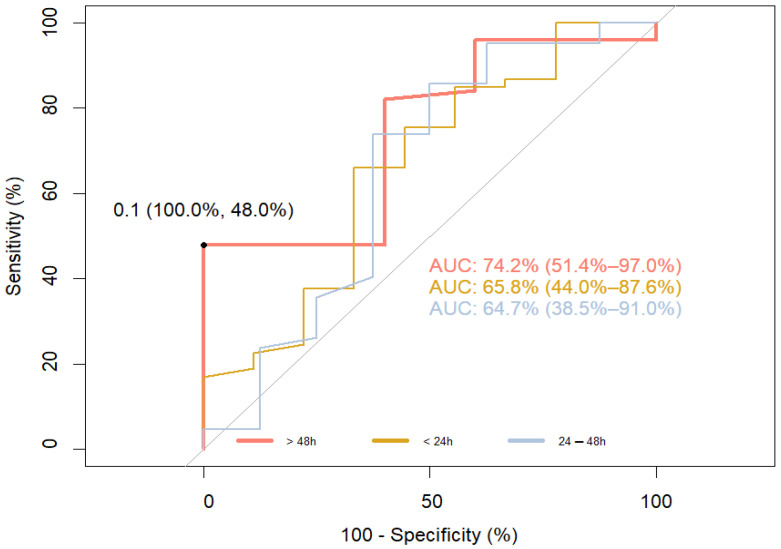
ROC curve to determine the sensitivity and specificity of S100B at different time points, predicting the short-term outcome measured as “no improvement” (delta NIHSS ≤ 0) and “improvement” (delta NIHSS > 0). The bootstrap method was used to determine confidence intervals. The Youden Index was used for setting optimal thresholds.

For the ROC curve to determine the sensitivity and specificity of S100B at different time points predicting the short-term outcome measured as “no improvement” (delta NIHSS ≤ 0) and “improvement” (delta NIHSS > 0), the AUC was 74.2% (51.4–97.0%) for time point >48 h (sensitivity 48.0, specificity 100.0%). For other sampling points, the AUC was 65.8% (<24 h) and 64.7% (24–48 h), respectively (Figure 2).

**Figure 3:**
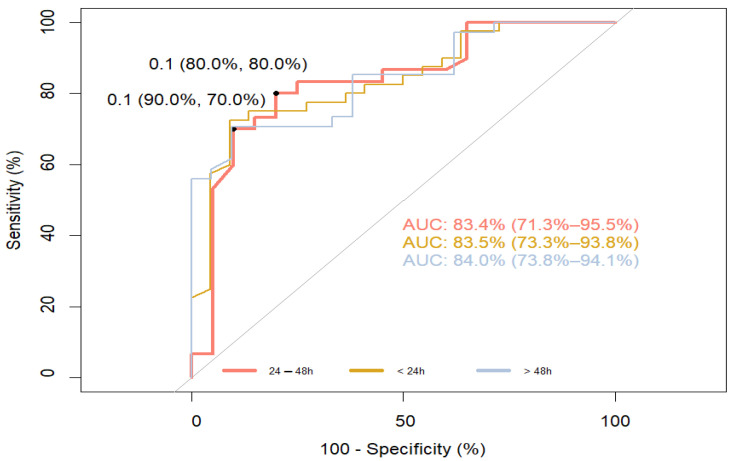
ROC curve to determine the sensitivity and specificity of S100B at different time points, predicting the long-term outcome measured as “good” (MRS 0–2) and “poor” (MRS ≥ 3). The Youden Index was used for setting optimal thresholds.

For the ROC curve to determine the sensitivity and specificity of S100B at different time points, predicting the long-term outcome measured as “good” (MRS 0–2) and “poor” (MRS ≥ 3), the AUC was 83.4% (71.3–95.5%) for time point 24–48 h (sensitivity 80.0%, specificity 80.0%). For other sampling points, the AUC was 83.5% (73.3–93.8%) (<24 h) (sensitivity 80.0%, specificity 80.0%) and 84.0% (73.8–94.1%) (24–48 h), respectively (Figure 3).

According to the ROC curve analysis, we used the cut-off 0.100 μg/L and then categorized the subjects into two groups. The first group included subjects with a serum S100B level of 0.100 μg/L or less, and the second group consisted of subjects with a S100B level of 0.100 μg/L or above. We found out that this cut-off threshold was useful in defining groups with better prognoses in terms of early and late disability. Results of this analysis are provided in Table 4 and Table 5.

Analysis of variance did not reveal any statistically significant differences in the mean levels of S100B between various subtypes of ischemic stroke—LAA, SAO, CE, and SUC (stroke of undetermined etiology), with the *p*-values of 0.911, 0.369, 0.262, and 0.693, respectively. 

No statistically significant differences were also revealed in the mean levels of S100B between patients who had undergone recanalization (thrombolytic therapy, mechanical thrombectomy, or the combination of both) and patients treated conservatively. The *p*-value was 0.649, 0.245, and 0.837, respectively (see Figures S1–S7, Supplementary Files).

## 4. Discussion

The main finding supporting the validity of our study is that serum S100B levels in patients with ischemic stroke, measured on day 1, 2 and 3, are strongly correlated with the short-term functional outcomes determined with the NIHSS on day 9 after the stroke, as well as with the long-term outcomes, which were assessed with the MRS after 3 months (Figure 1 and Figure 3).

Our interest in the S100B protein stems from several reasons. Despite the development of diagnostic methods, there is still no way to continuously, non-invasively, and inexpensively monitor the changes in brain tissue that occur as a result of ischemia. S100B, unlike other proteins in the S100 family, is more specific to brain tissue and appears in the blood when the brain is damaged rather than other organs [21]. Perfusion is necessary for this protein to appear in the blood, so its presence is not related to the infarct core but to the penumbra and the developing cytotoxic cerebral edema. Moreover, once released from the cell, it promotes inflammation and increases cytotoxic edema. These properties, further supported by clinical studies, make S100B an excellent marker for stroke severity regarding active metabolic responses [13,14,22]. Given the short half-life of this protein and renal clearance, serum S100B levels illustrate the course of ischemic stroke in real-time [15,16]. It seems that these suppositions are in line with clinical observations. Elting et al. compared changes in S100B levels in patients with ischemic stroke, transient ischemic attack (TIA), and traumatic brain injury (TBI). In patients with ischemic stroke, peak levels of S100B were most often observed on day 3 or 4 after the stroke (during the formation of cytotoxic edema), whereas in patients with TBI, peak levels were observed on day 1 or 2 after the injury [15]. The same results were obtained by Weglewski et al. who studied 57 patients and observed peak levels of S100B on day 3 after ischemic stroke [23]. An important question is whether short- and long-term functional outcomes can be predicted based on the changes in S100B levels. Our study revealed that S100B levels measured on days 1, 2 and 3 were significantly correlated with the NIHSS scores on day 9 and the MRS scores after 3 months (Table 3). In addition, ROC curve analysis confirmed a high sensitivity and specificity of S100B in predicting functional outcomes (Figure 1 and Figure 3). The power computation for ROC curves was confirmed by the results obtained for the odds ratio. These results do not fully match those obtained by other researchers. Selcuk et al. searched for correlations between S100B levels measured on days 1, 3, 5 and the MRS score after 1 month. They revealed only a weak correlation with S100B measured on day 3. However, it should be remembered that their study was conducted on a very small group of patients (*n* = 26), and the time points for the functional assessment with the MRS were different from those in our study [24]. Abdel et al. determined the serum S100B levels in a group of 40 patients on day 1 and 3 and then correlated the results with the NIHSS and MRS on day 14. They found a strong correlation on day 3, but not on day 1. The AUC calculated based on the ROC curve was characterized by a high sensitivity and specificity (91.0% and 80.0%, respectively) and was similar to our results [25]. The lack of correlation on day 1 may be due to the methodology as the measurement of the S100B level can vary in time up to several minutes. Presently, there are no universally adopted standards regarding the time for collecting samples. Moreover, researchers use different time points to assess the outcome and different clinical scales. This can be confirmed by the results obtained by Singapore researchers because their study was designed similarly to ours. In order to determine S100B levels, blood was collected on days 1 and 3, and the assessment with the MRS was performed after 1 and 3 months. A strong correlation between S100B levels and the functional outcome after 3 months was revealed. A very interesting finding of that study was the relationship between the functional outcome and the rate of the decrease of S100B levels over time [26]. Branco et al. studied, in the same way as we did, a group of 131 patients only with ischemic stroke [27]. They determined biomarker levels (CRP, D-dimer, and fibrinogen) on admission, while blood was collected 48 h later to measure S100B levels. The results obtained were compared with the MRS scores after 48 h and 12 weeks following the stroke. The authors revealed an evident correlation between S100B levels and the MRS scores after 12 weeks. Their results are in line with our findings. Also, the cut-off point for S100B in that study (140 ng/L) was the same as ours. However, no association was found between the other biomarkers tested and the functional outcome. Retnaningsih et al. arrived at interesting findings [28]. In their study conducted on 42 patients with ischemic stroke, they measured S100B levels on day 3 after the stroke. Stroke severity was assessed with the NIHSS on days 3 and 7. Then, the patients were divided into two groups: with improvement according to the NIHSS score and without improvement. The ROC curve was analyzed to establish the cut-off point for S100B. This parameter was employed to determine the odds ratio for clinical improvement and, consequently, for a good functional outcome. For the cut-off point at the level of 608 ng/mL and below, the odds ratio for the improved outcome was six-fold higher than for the value of S100B above 608 ng/mL (26). In our study, we also attempted to determine the odds ratio for clinical improvement. Our results in this regard were not satisfactory due to the small number of measurements (Figure 2). However, our calculations for the beneficial outcome showed the strength of the ROC curve (Table 4 and Table 5). Although we did not reveal significant differences in S100B levels between patients who had recanalization performed, in our view, this is not inconsistent with the latest study by Luger et al. In their study involving 171 patients, they demonstrated that the S100B level assessed on the second day following thrombectomy is an independent prognostic factor in the long-term functional outcome of ischemic stroke. Unfortunately, they did not compare the results they obtained with the group without recanalization [29]. In another study, Hawash et al. assessed the prognostic usefulness of S100B in a group of 80 patients with ischemic stroke. Their analyses indicate that S100B alone is a significant prognostic factor, but the performed thrombolysis does not influence its prognostic strength [17]. Our results, similarly to the aforementioned studies, suggest that the statistical strength of S100B in determining long-term functional outcome in ischemic stroke is similar for all patients, irrespective of the introduced treatment (Figures S5–S7). As in the case of treatment methods, we also did not find significant correlations between ischemic stroke subtypes and S100B levels. This is intriguing because S100B is an indicator of the size of the infarct core, and so we had expected to find at least a correlation between S100B levels and ischemic stroke caused by large artery atherosclerosis (LAA). Nevertheless, other researchers obtained similar results. Foerch et al. assessed the usefulness of S100B in predicting malignant course of infarction resulting from the occlusion of the middle cerebral artery (MCA). They revealed that the occlusion of MCA is as frequent in the course of LAA as in the cardio embolism subtype [30]. Analogous results were obtained by Yoruk Hazar et al., who examined 50 patients with ischemic stroke and compared the results with those of healthy volunteers [18]. Researchers from Singapore arrived at even more far-reaching conclusions. Sakdejayont et al. revealed that the level of S100B allows for the determination of long-term functional outcomes and severity of the course of ischemic stroke, irrespective of its mechanism. S100B levels were measured before the treatment was commenced (thrombolysis, thrombectomy, antiplatelet therapy) and after 72 h. The difference between these levels was also determined. Stroke severity was established based on the NIHSS score, and the outcome was measured with the Ranking Scale after 30 and 90 days. Although their study group had different characteristics than our study (small artery occlusion was the dominating subtype), the relation of groups with LAA occlusion and cardio embolism vs. small artery occlusion and EUS was similar. The conducted analyses revealed that the S100B level after 72 h and the change in the levels between the measurement points are the optimal prognostic factors for the adverse outcome irrespective of the stroke type [26]. This seems to be easily explained: prognosis depends on the size of the ischemic area and not on the stroke etiology. We believe that our results, which confirm those obtained by other researchers, will consolidate knowledge in this area (Figures S1–S4). We are aware of the limitations of our study. A major drawback is the lack of a control group due to financial constraints. The small group of patients included in the study did not allow us to conduct reliable statistical analyses to independently evaluate other risk factors affecting the course of the disease. In particular, we regret that we were unable to determine the usefulness of S100B in monitoring permanent recanalization because this might potentially make this molecule a cheap and non-invasive marker.

## 5. Conclusions

Ultimately, we suggest that serum S100B protein levels can predict the course of ischemic stroke in the context of short- and long-term functional outcomes.

## Figures and Tables

**Table 1:** Baseline characteristic and stroke risk factors.

Factor	Total Patients (*n* = 65)
Sex (female)	34 (52.31%)
Age (years)	67.43 ± 13.08 *
Risk factors:	
Hypertension	45 (69.23%)
Diabetes	23 (35.38%)
Dyslipidemia	26 (40.00%)
Atrial fibrillation	28 (43.08%)
Smoking	32 (49.23%)
Thrombectomy only	5 (7.70%)
Thrombolysis only	20 (30.77%)
Thrombolysis and thrombectomy	10 (15.40%)
TOAST:	
Large artery atherosclerosis (LAA)	13 (20.00%)
Small artery occlusion (SAO)	12 (18.46%)
Cardio embolism (CE)	20 (30.77%)
Stroke of undetermined etiology (SUE)	20 (30.77%)
Stroke of other determined etiology	0 (0.00%)

* mean and SD.

**Table 2:** Comparison of NIHSS groups.

NIHSS on Admission	NIHSS on Day 9		Total
	0–8	>8	
0–8	38	0	38
>8	15	12	27
Total	53	12	65
*p* < 0.001	McNemary test (A/D)		

**Table 3:** Correlation between S100B < 24 h and 24–48 h and 48–72 h with NIHSS, delta NIHSS, and MRS.

		NIHSS on Day 9	Delta NIHSS	MRS after 3 Months
S100B < 24 h	R(p)	0.534 (0.000)	−0.039 (0.761)	0.620 (0.000)
S100B 24 h–48 h	R(p)	0.631 (0.000)	0.127 (0.899)	0.657 (0.000)
S100B > 48 h	R(p)	0.517 (0.000)	−1.176 (0.245)	0.617 (0.000)

**Table 4:** OR for a chance to achieve a better functional result—MRS 0–2 after 3 months.

	Cut-Off, μg/L	MRS 0–2 OR	*p*
S100B < 24 h	0.1	4.78 (95% CI: 1.47; 15.55)	0.009
S100B 24 h–48 h	0.1	OR = 9.35 (95% CI: 3.04; 28.77)	0.001
S100B 48 h–72 h	0.1	OR = 4.26 (95% CI: 1.42; 12.8)	0.01

**Table 5:** OR for a chance to achieve a better functional result—NIHSS 0–8 on day 9.

	Cut-Off	NIHSS 0–8 OR	*p*
S100B < 24 h	0.1	13.51 (95% CI: 3.24; 56.36)	0.001
S100B 24 h–48 h	0.1	15.35 (95% CI: 4.06; 58)	0.001
S100B 48 h–72 h	0.1	4.48 (95% CI: 1.12; 17.92)	0.034

## Data Availability

The data that support the findings of this study are available on request from the corresponding author.

## Supplementary Materials

The following supporting information can be downloaded at https://www.mdpi.com/article/10.3390/jpm14010080/s1, Figure S1. Distribution of S100B levels over time according to stroke subtype—LAA. Figure S2. Distribution of S100B levels over time according to stroke subtype—SAO. Figure S3. Distribution of S100B levels over time according to stroke subtype—CE. Figure S4. Distribution of S100B levels over time according to stroke subtype—SUE. Figure S5. Distribution of S100B levels over time according to stroke treatment—thrombolysis vs. no recanalisation. Figure S6. Distribution of S100B levels over time according to stroke treatment—thrombectomy vs. no recanalisation. Figure S7. Distribution of S100B levels over time according to stroke treatment—thrombolysis with thrombectomy vs. no recanalisation.
