# Supplementary material for: Predicting Short- and Long-Term Functional Outcomes Based on Serum S100B Protein Levels in Patients with Ischemic Stroke
Source: J Pers Med. 2024 Jan 10;14(1):80. doi: 10.3390/jpm14010080 (PMC10817633; doi:10.3390/jpm14010080)
Supplement: Supplementary file 1 [file jpm-14-00080-s001.zip › Figure S6.pdf]

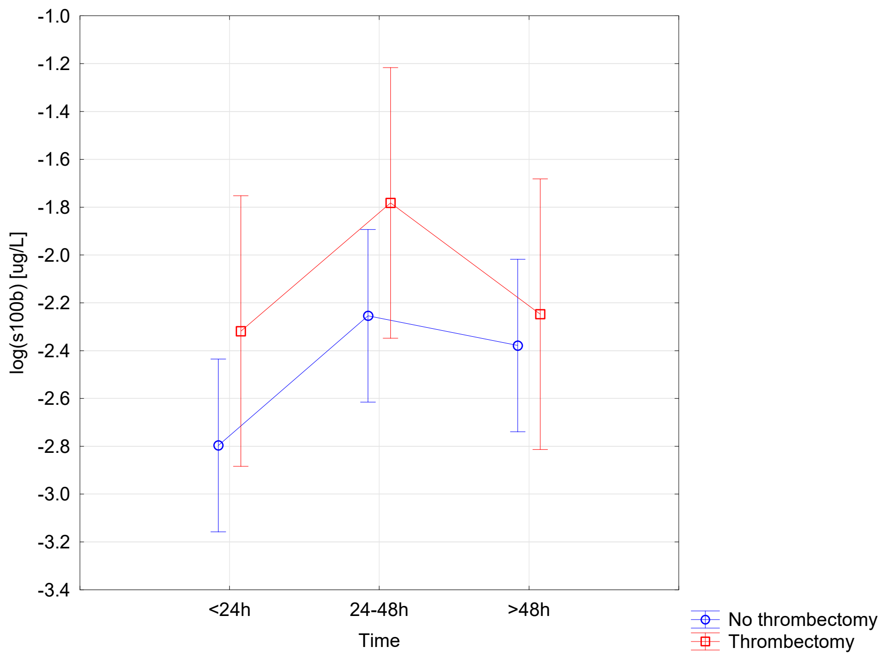

Figure S6. Distribution of S100B levels over time according to stroke treatment - thrombectomy vs no recanalisation.
