# Supplementary figures and images for: Predicting Short- and Long-Term Functional Outcomes Based on Serum S100B Protein Levels in Patients with Ischemic Stroke
Source: J Pers Med. 2024 Jan 10;14(1):80. doi: 10.3390/jpm14010080 (PMC10817633; doi:10.3390/jpm14010080)

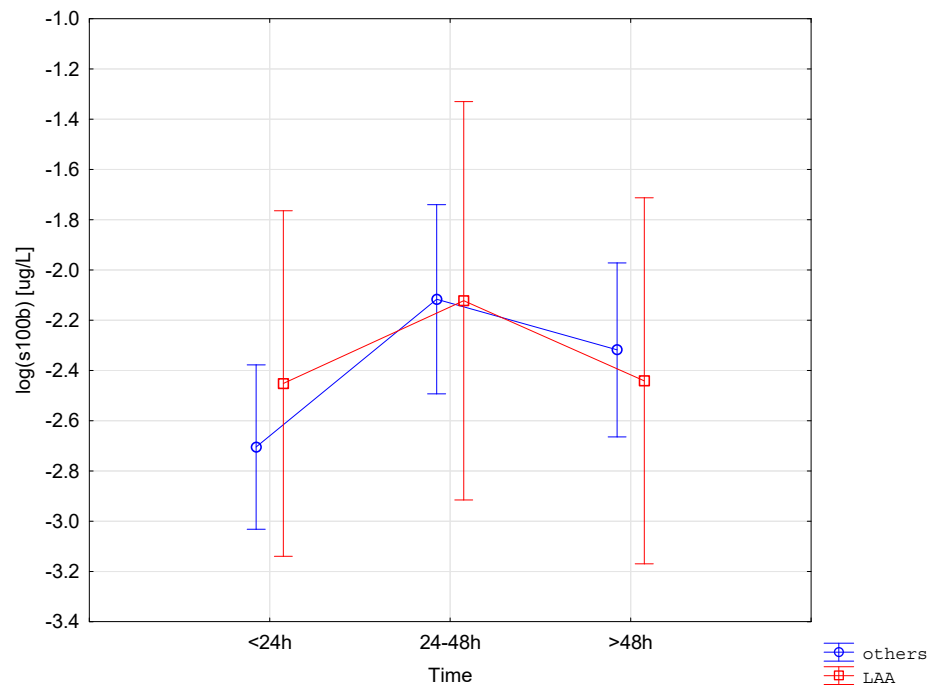

Figure S1. Distribution of S100B levels over time according to stroke subtype – LAA

Supplement: Supplementary file 1 [file jpm-14-00080-s001.zip › Figure S1.pdf]

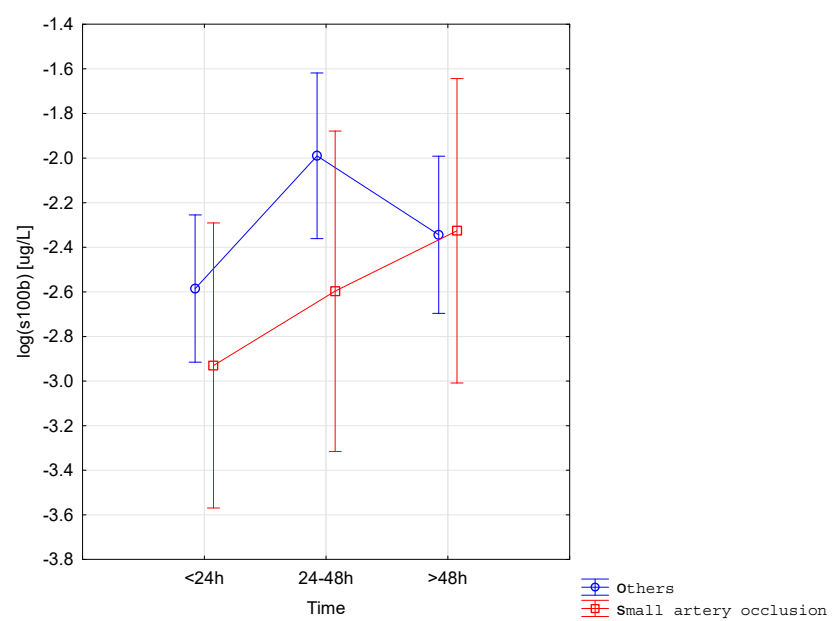

Figure S2. Distribution of S100B levels over time according to stroke subtype – SAO

Supplement: Supplementary file 1 [file jpm-14-00080-s001.zip › Figure S2.pdf]

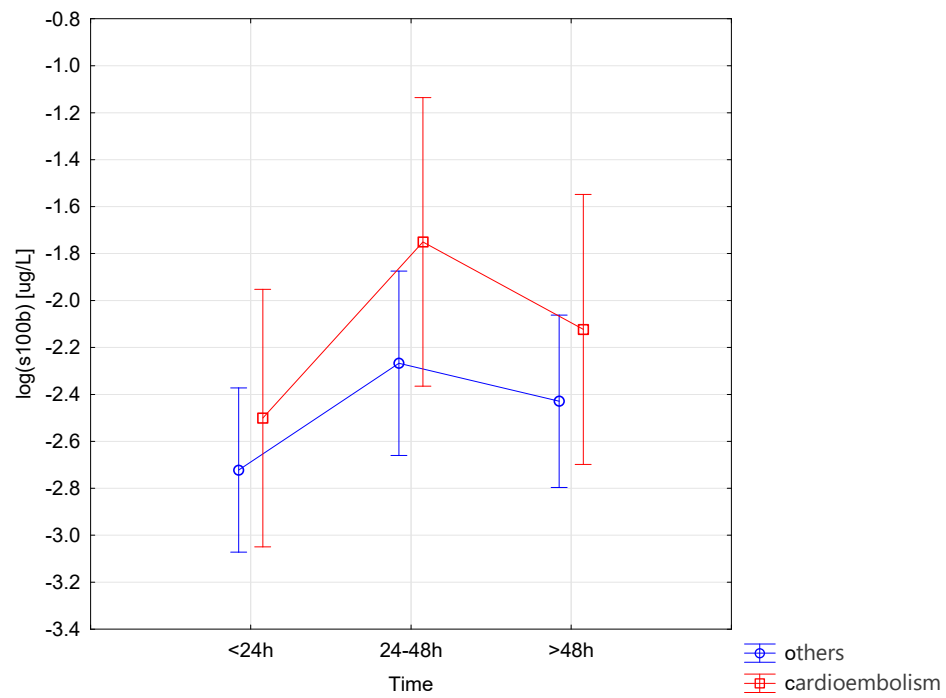

Figure S3. Distribution of S100B levels over time according to stroke subtype – CE

Supplement: Supplementary file 1 [file jpm-14-00080-s001.zip › Figure S3.pdf]

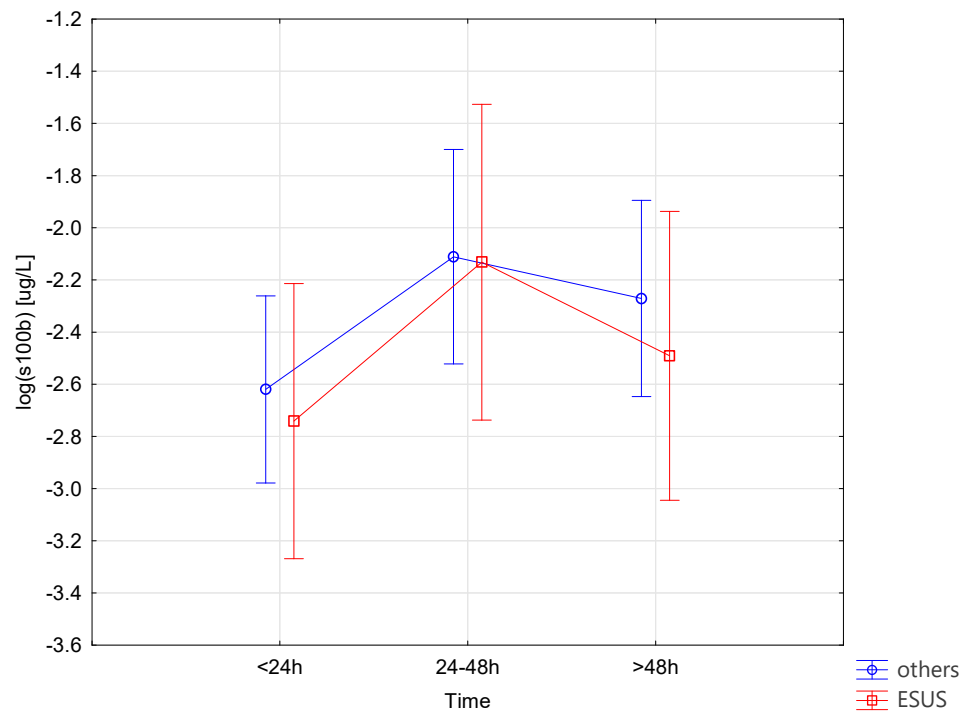

Figure S4. Distribution of S100B levels over time according to stroke subtype – SUE

Supplement: Supplementary file 1 [file jpm-14-00080-s001.zip › Figure S4.pdf]
